# Rationale and design of a proof-of-concept trial investigating the effect of uninterrupted perioperative (par)enteral nutrition on amino acid profile, cardiomyocytes structure, and cardiac perfusion and metabolism of patients undergoing coronary artery bypass grafting

**DOI:** 10.1186/1749-8090-6-36

**Published:** 2011-03-25

**Authors:** Marlieke Visser, Mariska Davids, Hein J Verberne, Wouter EM Kok, Hans WM Niessen, Lenny MW van Venrooij, Riccardo Cocchieri, Willem Wisselink, Bas AJM de Mol, Paul AM van Leeuwen

**Affiliations:** 1 Department of Cardiothoracic Surgery, Academic Medical Center University of Amsterdam, Amsterdam, The Netherlands; 2 Department of Surgery, VU University Medical Center, Amsterdam, The Netherlands; 3 Department of Clinical Chemistry, VU University Medical Center, Amsterdam, The Netherlands; 4 Department of Nuclear Medicine, Academic Medical Center University of Amsterdam, Amsterdam, The Netherlands; 5 Department of Cardiology, Academic Medical Center University of Amsterdam, Amsterdam, The Netherlands; 6 Department of Pathology and Cardiac Surgery, VU University Medical Center, Amsterdam, The Netherlands; 7 iCaR-VU, VU University Medical Center, Amsterdam, The Netherlands

## Abstract

**Background:**

Malnutrition is very common in patients undergoing cardiac surgery. Malnutrition can change myocardial substrate utilization which can induce adverse effects on myocardial metabolism and function. We aim to investigate the hypothesis that there is a disturbed amino acids profile in the cardiac surgical patient which can be normalized by (par)enteral nutrition before, during and after surgery, subsequently improving cardiomyocyte structure, cardiac perfusion and glucose metabolism.

**Methods/Design:**

This randomized controlled intervention study investigates the effect of uninterrupted perioperative (par)enteral nutrition on cardiac function in 48 patients undergoing coronary artery bypass grafting. Patients are given enteral nutrition (n = 16) or parenteral nutrition (n = 16), at least two days before, during, and two days after coronary artery bypass grafting, or are treated according to the standard guidelines (control) (n = 16). We will illustrate the effect of (par)enteral nutrition on differences in concentrations of amino acids and asymmetric dimethylarginine and in activity of dimethylarginine dimethylaminohydrolase and arginase in cardiac tissue and blood plasma. In addition, cardiomyocyte structure by histological, immuno-histochemical and ultrastructural analysis will be compared between the (par)enteral and control group. Furthermore, differences in cardiac perfusion and global left ventricular function and glucose metabolism, and their changes after coronary artery bypass grafting are evaluated by electrocardiography-gated myocardial perfusion scintigraphy and ^18^F-fluorodeoxy-glucose positron emission tomography respectively. Finally, fat free mass is measured before and after intervention with bioelectrical impedance spectrometry in order to evaluate nutritional status.

**Trial registration:**

Netherlands Trial Register (NTR): NTR2183

## Background

Malnutrition is very common in patients undergoing cardiac surgery as well as other types of surgery. For example, in a population of cardiac and abdominal surgical patients, respectively 10-25% [1,2] and 44% [3] was malnourished. Malnutrition is an independent risk factor resulting in more complications, and increased mortality rates, length of hospital stay and costs [1-3]. The lack of optimal nutrition can change myocardial substrate utilization which can have adverse effects on myocardial metabolism such as adenosine triphosphate (ATP) production and utilization [4]. For that reason, malnutrition might be an underlying risk factor for the perioperative cardiac complications observed in patients undergoing non-cardiac surgery [5]. In addition to cardiac complications, the lack of optimal nutrition can induce nutrient deficiencies which in turn can lead to the impairment of the immune system [6]. It is still common practice that patients receive only clear fluids during the period prior to surgery and the day after surgery leading to starvation of the patient over a longer period of time. As this occurs, glycogen reserves that last only a few hours will deplete with the result that further fasting induces gluconeogenesis. As this gluconeogenesis mainly depends on catabolism of body proteins it furthers the negative effects of malnutrition. We hypothesize that avoidance of malnutrition and starvation can improve cardiac metabolism and function, and might prevent protein catabolism, which would be beneficial for both cardiac and non-cardiac surgical patients.

Nitric oxide (NO), created from the amino acid arginine, is a regulator of cardiac and vascular function. However, the actions of NO can be disturbed by elevated levels of the NO synthase (NOS) inhibitor, asymmetric dimethylarginine (ADMA), a condition reported in patients with failing hearts [7]. Moreover, ADMA has been indicated as marker of circulatory function and as predictor of outcome in patients with cardiac dysfunction [7,8]. As NO availability might be reflected by the ratio between substrate (arginine) and inhibitor (ADMA), the negative effects of ADMA might be relieved by supplementation of arginine. However, the effect of arginine supplementation is complicated as studies have shown both positive and negative results in critically ill patients [7]. Probably, the ratio between arginine and ADMA might play a role as NO availability needs to be perfectly balanced in order to guarantee proper cardiac contraction and vascular dynamics. We hypothesize that nutrition containing arginine is a safe method that might improve the whole amino acid profile in patients with cardiac dysfunction.

Therefore, in this proof-of-concept study, we aim:

(1) To evaluate the effect of uninterrupted perioperative (par)enteral nutrition supplementation versus no supplementation on amino acid profile and cardiomyocytes structure in patients undergoing coronary artery bypass grafting (CABG).

(2) To study the effect of uninterrupted perioperative (par)enteral nutrition supplementation versus no supplementation on myocardial perfusion, left ventricle function and glucose metabolism before and after CABG.

(3) To study the effect of uninterrupted perioperative (par)enteral nutrition supplementation versus no supplementation on fat free mass (FFM, as a marker of nutritional status) before and after CABG.

## Methods/Design

### Design

This is a randomized controlled intervention study. The research protocol of this clinical trial (NTR2183, EudraCTnr 2009-017812-33) has been reviewed and approved by the Medical Ethical Committee of the Academic Medical Center of the University of Amsterdam (AMC) (MEC 09/304) and the Competent Authority of the Netherlands (Centrale Commissie Mensgebonden Onderzoek) (NL28231.018.09).

### Participants

Patients undergoing cardiac bypass surgery are selected to study the effects of (par)enteral nutrition on human cardiac tissue. In order to prevent cardioplegic effects on cardiomyocytes, selected patients are undergoing an off-pump CABG-procedure. Forty-eight patients with stable anginal complaints planned for an elective CABG who meet all inclusion criteria and do not have any of the exclusion criteria (Table 1) will be randomized by computer-generated block randomization (each block including six patients) to one of the three study groups.

**Table 1:** Inclusion & exclusion criteria

Inclusion criteria:	
	•Undergoing off-pump CABG-surgery
	•Age 18 till 80 years
Exclusion criteria:	
	•Combined valve and CABG procedure
	•Age <18 and ≥ 80 years
	•Diabetes mellitus type I
	•Pregnancy
	•Renal insufficiency defined as creatinine > 95 μmol/L for women and > 110 μmol/L for men
	•Liver insufficiency defined as ALAT > 34 U/I for women and > 45 U/I for men

ALAT, alanine aminotransferase; CABG, coronary artery bypass grafting;.

### Setting

**Figure 1:**
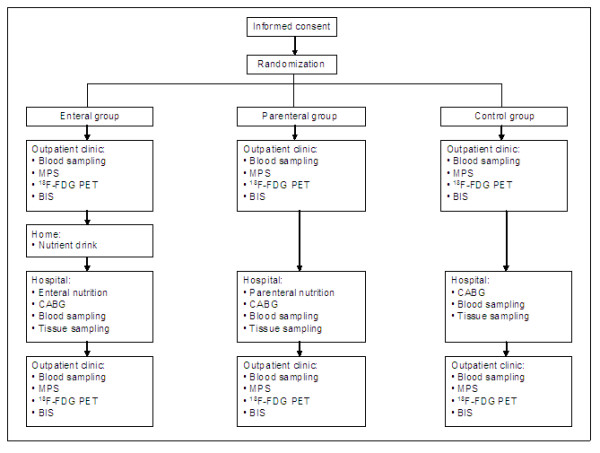
**Flow chart of study protocol**. BIS, bioelectrical impedance spectrometry; CABG, coronary artery bypass grafting; ^18^F-FDG PET, ^18^F-fluorodeoxy-glucose positron emission tomography; MPS, myocardial perfusion scintigraphy.

Figure 1 depicts a flow chart of the study protocol. The study is currently performed at the department of cardio-thoracic surgery of the AMC. Patients are informed about the study by a cardiologist and nutritionist who hand over the information letter as well as the informed consent form. A patient is included in the study when the informed consent form is signed. Subsequently, the patient can be randomized to one of the three groups: the "enteral group" (n = 16), the "parenteral group" (n = 16), or the control group (n = 16). During visits at the hospital outpatient clinic (approximately two weeks before surgery), patients are subjected to baseline measurements including blood sampling, measurement of body weight and height, bioelectrical impedance spectrometry (BIS), ECG-gated myocardial perfusion scintigraphy (MPS) and a ^18^F-fluorodeoxy-glucose positron emission tomography (^18^F-FDG PET) scan (Table 2). As part of the preoperative evaluation, patients will also undergo preoperative assessments by a cardiothoracic surgeon and an anesthesiologist.

**Table 2:** Study schedule

Days	Before								CABG			After			
	**-14**	**-7**	**-6**	**-5**	**-4**	**-3**	**-2**	**-1**	**0 ** **before**	**0 ** **start**	**0 ** **end**	**1**	**2**	**3**	**>21**

Informed consent	E,P,C														
Randomization	E,P,C														
Blood sampling	E,P,C								E,P,C	E,P,C	E,P,C	E,P,C		E,P,C	E,P,C
MPS	E,P,C														E,P,C
^18^F-FDG PET	E,P,C														E,P,C
BIS	E,P,C														E,P,C
Cardiac tissue										E,P,C	E,P,C				
Aortic tissue											E,P,C				
Nutrient drink		E	E	E	E										
Hospital admission						E,P		C							
Nutrition						E,P	E,P	E,P	E,P	E,P	E,P	E,P	E,P		

BIS, bioelectrical impedance spectrometry; C, control group; CABG, coronary artery bypass grafting; E, enteral group; ^18^F-FDG PET, ^18^F-fluorodeoxy-glucose positron emission tomography; MPS, myocardial perfusion scintigraphy; P, parenteral group.

Patients allocated to the enteral and parenteral group will be admitted at the hospital three days before surgery (Table 2). Patients allocated to the control group will be admitted one day before surgery.

Approximately three weeks after surgery patients will visit the outpatient clinic for measurements including blood sampling, measurement of body weight, BIS, MPS and ^18^F-FDG PET. In addition, as part of the routine clinical postoperative care patients will be seen by a cardiothoracic surgeon.

### Nutrition and administering devices

#### Enteral nutrition

During the four days before hospital admission, the enteral group will take 125 ml per day of a nutrient drink (Nutridrink Compact, Nutricia, Zoetermeer, The Netherlands) consisting of proteins, carbohydrates, fats, vitamins and minerals (Table 3). When admitted to the hospital, patients in the enteral group will receive a solution containing amino acids (PeptoPro, DSM, Delft, The Netherlands), carbohydrates (Fantomalt, Nutricia, Zoetermeer, The Netherlands), and vitamins and minerals (Phlexy-Vits, SHS International Ltd., Liverpool, United Kingdom) which will be prepared at the hospital each day. An amount of 1050 ml of the enteral nutrition will be given during 24 hours. This nutrition will be given two days before, during and two days after CABG by a computerized guidance system-placed nasoduodenal tube (Cortrak^®^, Viasys Healthcare, Wheeling, IL, USA). At the morning of surgery the position of the duodenal tube is verified. Patients are permitted to eat and drink in addition to their supplemental nutrition.

**Table 3:** Composition of enteral and parenteral nutrition

	Enteral group		Parenteral group
	Drink (at home) per day	Nutrition (at hospital) per day	Nutrition (at hospital) per day
Volume (ml)	125	1050	1250
Amino acids (g)	12	80.5	40
Carbohydrates (g)	37.1	95	80
Fat (g)	11.6	1.5	50
Energy (kcal)	300	745	955
Vitamins and minerals	Yes	Yes	Yes

#### Parenteral nutrition

Patients in the parenteral group will receive 1250 ml of nutrition (Nutriflex Lipid peri, B.Braun, Oss, The Netherlands) containing amino acids, lipids and glucose. An amount of 1250 ml of the amino acid infusion (840 mOsm/L) will be given in 24 hours for 5 days (Table 3). In addition, vitamins (Cernevit, Baxter, Utrecht, The Netherlands) and trace elements (Nutritrace, B.Braun, Oss, The Netherlands) will be added to the parenteral nutrition. This nutrition will be given two days before, during and two days after CABG. Patients are permitted to eat and drink in addition to their supplemental nutrition.

#### Controls

The control group follows the standard protocol of the department of cardio-thoracic surgery of the AMC allowing patients to eat and drink until six hours before surgery. The day after surgery, this standard protocol prescribes a (clear) liquid diet. On the second day after surgery patients are recommended a normal diet.

### Outcome measures

The main study outcomes are amino acid profile and cardiomyocytes structure at the time of cardiac surgery. Amino acid profile will be studied in blood plasma and cardiac tissue and includes determining the concentrations of all amino acids, ADMA and symmetric dimethylarginine (SDMA, ADMA's isomer that lacks direct NOS inhibitory activity). In addition, the arginine/ADMA ratio (an indicator of potential NO production) will be calculated. In cardiac tissue, also the activity of dimethylarginine dimethylaminohydrolase (DDAH, an enzyme which degrades ADMA) and arginase (an enzyme which metabolizes arginine) are measured. Cardiomyocytes structure will be assessed by histological analysis, immuno-histochemistry, and by electron microscopy.

The secondary study outcomes are cardiac perfusion, left ventricular function and cardiac glucose metabolism. ECG-gated MPS will be used for the measurement of cardiac perfusion and left ventricular function. Cardiac glucose metabolism will be measured with ^18^F-FDG PET.

BIS measured FFM will be used as parameter of nutritional status. A high FFM is related to better nutritional status and improved post-surgical outcome [9]. Other outcome parameters are cardiac muscle damage and signs of failure as measured by blood plasma biomarkers (Troponin T, CK-MB, and NT-proBNP). Blood plasma will be stored for future study of metabolic switch biomarkers. Baseline characteristics (including European System for Cardiac Operation Risk Evaluation score (EuroSCORE) [10], and unintended weight loss) will be recorded, as well as clinical parameters (intensive care unit (ICU) stay, length of hospital stay, time of mechanical ventilation, organ failure, infections, bleeding, and postoperative mortality). Finally, the concentration of ADMA will be measured in a sample of the aortic wall to investigate the relation between tissue ADMA concentrations in the aorta, and both intracellular concentrations from peripheral blood mononuclear cells (PBMC) and plasma levels.

### Blood and tissue samples

Blood sampling will be done at baseline (approximately two weeks before CABG during a visit at the hospital outpatient clinic), at the day of surgery (once before and twice during surgery), at the first and third day after surgery, and approximately three weeks after surgery (during a visit at the hospital outpatient clinic) (Table 2).

During surgery, two tissue samples of the appendix of the right atrium will be taken by the surgeon. One sample will be taken prior to the start of the anastomosic connection of the bypass graft, and one sample at the end of the procedure before closing of the pericardium. Half of each sample is placed in an aluminum box which will be immediately frozen in liquid nitrogen and stored at -80°C until the amino acid profile is analyzed. The other half of each sample is immediately placed in formalin and will be analyzed within two weeks for the assessment of cardiomyocytes structure. In addition, a sample of aortic tissue is taken by the surgeon at the end of the CABG-procedure which becomes available due to fixation of the proximal anastomosis needed for the bypass and is in routine clinical practice discarded. The sample is immediately frozen in liquid nitrogen and stored at -80°C until the ADMA concentration is measured.

### Nuclear medicine imaging techniques

An ECG-gated MPS and a ^18^F-FDG PET scan are performed at baseline (approximately two weeks before CABG) and more than three weeks after CABG. Stress and rest myocardial perfusion scintigraphy (with single-photon emission computed tomography (SPECT)) is performed with ^99 m^Tc labeled Tetrofosmin. Symptom limited exercise is the preferred stress modality. Pharmacological vasodilatory stress with adenosine will be applied if there is an insufficient increase of heart rate (<85% age predicted maximal heart-rate) during physical exercise, in the presence of a left bundle branch block, or if the anti-anginal medication had not been adequately discontinued. Dobutamine stress testing is performed in patients with a contra-indication for adenosine. The type of stress test applied for MPS before surgery is maintained at the MPS post-surgery. ECG-gated image acquisition is performed for the assessment of parameters of left ventricular function (end-systolic and end-diastolic volumes and ejection fraction). Two experienced nuclear medicine physicians analyze the images in a total of 17 myocardial segments. Segments are scored with a 5-point scoring system (0 = normal; 1 = equivocal; 2 = moderate reduction; 3 = severe reduction; 4 = absent activity). Summed stress score (SSS) and summed rest score (SRS) are obtained by adding the scores of all segments of respectively stress and rest images. The summed difference score (SDS) is calculated by subtracting the SRS from the SSS. Reversible myocardial perfusion defects, indicative for inducible myocardial ischemia, are defined as SDS ≥3. Fixed defects, indicative for scarring are defined as a SRS-score of ≥3. The presence of either reversible or fixed defects is defined as the presence of any perfusion defect.

^18^F-FDG is a glucose analogue that after cellular uptake via the GLUT-4 transporter and phosphorylation by hexokinase is not further metabolized. Therefore the imaged concentration of ^18^F-FDG in the heart reflects its glucose metabolism. Patients are imaged after suppression of the free fatty acid metabolism by oral administration of acipimox. The glucose metabolism in the myocardium is analyzed by standardized uptake values, both regional as for the total myocardium.

### Bioelectrical impedance spectrometry

A BIS-measurement (BodyScout, Fresenius Kabi, 's-Hertogenbosch, The Netherlands) is performed at baseline and approximately three weeks after CABG for the assessment of body composition. The principle of the BIS is based on the conductance through body fluid of an electric current (5-800 μA, 5 kHz-1 MHz). The BIS measures the impedance at a range of frequencies from which the resistances of extra-cellular water and intracellular water are extrapolated. Resistance is measured on the right side while patients in supine position. Subsequently, FFM is calculated (FFM is linearly related to height^2^/body resistance) [11].

### Anesthetic and (post-)surgical procedures

Anaesthesia is induced with sufentanil 3 μg kg^-1 ^(Suftena^®^, Janssen-Cilag, Tilburg, The Netherlands) and propofolol 50-100 mg (Fresenius Kabi, Den Bosch, The Netherlands). Pancuronium bromide 0.1 mg kg^-1 ^(Pavulon^®^, Organon, Oss, The Netherlands) is given for muscle relaxation. Morphine 20 mg is given as a slow bolus injection before start of surgery. Anaesthesia is maintained with a continuous infusion of propofolol 2-5 mg kg^-1 ^h^-1^.

The off-pump technique is used for all patients. After a median full sternotomy, a few superficial and deep pericardial sutures are placed to facilitate cardiac displacement. During anastomosis, a suction-type mechanical stabilizer (Octopus 4.3, Medtronic, Minneapolis, MN, USA) is used to immobilize the target site of coronary artery. Distal myocardial perfusion is maintained using intracoronary shunt tube (Anastaflo, Edwards Lifescience, Irvine, CA, USA). The basic strategy for myocardial revascularization is in situ grafting of the internal thoracic artery to the left coronary system with complementary saphenous vein. Vein-to-aorta proximal anastomosis is performed using partial clamping or an anastomotic device.

After surgery, patients are admitted to the ICU and treated according to a standardized clinical protocol. Fluid administration consists of NaCl 0.9% and hydroxyethyl starch 6% of molecular weight 200 kDa (Haes-Steril, Fresenius Kabi, Den Bosch, The Netherlands).

### Blood laboratory analyses

In blood plasma, the concentrations of amino acids, ADMA and SDMA will be analyzed by high performance liquid chromatography (HPLC)/fluorescence as described previously [12,13]. Briefly, solid-phase extraction is used to isolate ADMA, SDMA and arginine, and subsequently all amino acids and ADMA and SDMA are converted into stable adducts by derivatization with ophthalaldehyde reagent containing mercaptopropionic acid. Derivatives are then separated by reversed-phase HPLC using isocratic elution and fluorescence detection.

Blood plasma, concentrations of Troponin T, CK-MB, and NT-proBNP will be analyzed with standard laboratory tests.

PBMC are isolated from whole blood by centrifugation after which cells are washed with PBS, are counted (Cell Dyn 4000, Abbott, Hoofddorp, The Netherlands), and lysed. Finally, the intracellular ADMA concentration in PBMC will be measured by HPLC/fluorescence.

### Tissue laboratory analysis

After homogenization of cardiac or aortic tissue (OMNI 2000 homogenizer, OMNI international Inc., Gainesville, Virginia, USA), concentrations of amino acids, ADMA and SDMA will be analyzed by HPLC/fluorescence. In cardiac tissue, also the activity of DDAH will be determined by measuring citrulline formation during incubation of tissue homogenates with an excess of ADMA. Furthermore, the activity of arginase will be determined by measurement of ornithine formation during incubation of tissue homogenates with an excess of arginine. Both citrulline and ornithine formation will be analyzed by HPLC/fluorescene.

### Tissue pathological evaluation

The cardiac tissue sample will be fixated in formaline. It then will be studied by histological, immuno-histochemical and ultrastructural analysis. For histological analysis, tissue samples will be stained with hematoxylin and eosin (HE) and Elastica van Gieson (EVG). Subsequently, cardiomyocytes diameter, thickness of the endocardium, level of fibrosis (interstitial, replaced and perivascular) and the percentage of fat tissue contribution (replacement and perivascular) will be analyzed. Periodic acid Schiff digested (PAS/D) stained tissue segments will be evaluated to detect and quantify glycogen stacking. Finally, the presence of iron and amyloid will be established by ferron and Congo-red staining respectively. The immuno-histochemical part of the study includes analysis/quantification of lymphocytes, macrophages, neutrophil granulocytes, myocytolysis, and pro-inflammatory vessel damage by antibodies CD45, CD68, myeloperoxidase (MPO), C3d, and carboxymethyl lysine (CML) respectively. Using electron microscopy, we will determine myofibril density, cytosolic glycogen, expanded sarcoplasmatic reticulum (as marker of cellular damage), amount of mitochondria, damage to mitochondria (stacking as reversible damage, protein dots as irreversible damage), and thickness of the basal membrane of capillaries.

All analysis in this study will be done by analysts that are blinded to group assignment.

### Baseline and Clinical characteristics

Unintended weight loss before surgery is defined as [(current weight) - (weight 1 month ago)] > 5% or [(current weight) - (weight 6 months ago)] > 10%. Clinical characteristics (including risk score, length of stay at the ICU and hospital, time of mechanical ventilation, organ failure, infections, and bleeding) are extracted from medical case notes and an electronic database. This database includes the risk score based on EuroSCORE [10]. Organ failure after intervention will be aggregated from the presence of cardiac damage defined as CK-MB isoenzyme ≥ 100 μg/L and/or acute renal failure defined as postoperative serum creatinine ≥ 200 μmol/L or as need for dialysis, and/or neurologic failure defined as cerebrovascular accident or peripheral neuropathy. Bleeding is defined as abdominal bleeding or need for reoperation because of bleeding, and infection is defined as respiratory tract infection, urinary tract infection, mediastinitis, sternal wound infection, leg wound infection, and other infections (such as phlebitis and rare cases of intra-abdominal and dermatologic conditions). Mortality is defined as mortality during the period from hospital admission until the postoperative visit at the outpatient clinic.

### Statistical analysis

The results of the enteral and parenteral group will be compared with results of the control group. Differences between the (par)enteral and control group will be analyzed with Chi-square tests for categorical variables, with unpaired t-tests for continuous variables, and with the Mann-Whitney U test for non-normally distributed data. Correlations will be analyzed with Pearson's correlation or with the Spearman rank correlation coefficient. Multiple linear and multiple logistic regression models will be used to determine if differences between groups can be explained by the effect of (par)enteral nutrition, by confounders or by both. A p-value of ≤ 0.05 will be considered statistically significant.

## Discussion

Malnutrition and starvation in surgical patients can have a negative impact on cardiac function and metabolism. We will investigate if this problem can be relieved by supplementation of uninterrupted perioperative enteral or parenteral nutrition. To the best of our knowledge, this is the first randomized controlled trial that examines the effect of uninterrupted (par)enteral nutrition on cardiac function in cardiac surgical patients. In this proof-of-concept study we will explore the hypothesis that there is a disturbed amino acids profile in the cardiac surgical patient and that our uninterrupted perioperative nutrition will normalize this profile with a subsequent improvement in cardiomyocytes structure, and in cardiac perfusion and metabolism. The results from this study will increase knowledge about the effect of nutrition and about avoiding starvation in cardiac surgical patients and thereby improving cardiac metabolism and function which might improve outcome. Additionally, as perioperative starvation is common practice in all surgical patients, and malnutrition might be an underlying risk factor for the perioperative cardiac complications in non-cardiac surgeries, the results of this study will be valuable for the treatment of all surgical patients.

### Previous studies

Randomized controlled trials in humans in which arginine [14-16], aspartate [17], or glutamate [18] was administered, have shown improved cardiac flow [15,16], cardiac function (measured as plasma troponine T, creatine kinase (CK), and CK-MB) [14,17,18] and/or cardiac metabolism (measured as myocardial acidosis, ATP and lactate in myocardial biopsies) [17,18]. In animal studies, amino acid supplementation minimized cardiomyocytes apoptosis probably by increasing ATP production and myocardial oxygen consumption [19], by reducing myocardial ischemic damage, and by increasing diastolic pressure [20]. Parenteral amino acid supplementation increased esophageal core temperature, shortened duration of postoperative mechanical ventilation, ICU stay and hospitalization, and speeded tracheal extubation in patients undergoing CABG [21]. Enteral nutrition in cardiac surgical patients, repleted cardiomyocytes with nutrients, improved left ventricular end-diastolic volume before surgery [22], improved preoperative host defense, reduced the number of postoperative infections, and preserved renal function [23].

The results of the aforementioned studies show favorable effects of nutrition on cardiac function. However, the effect of uninterrupted perioperative supplementation of amino acids, glucose, vitamins and minerals on cardiac amino acid profile, cardiomyocytes structure, cardiac perfusion, left ventricular function and metabolism of cardiac surgical patients have never been investigated.

### Rationale for nutrients and administration devices

The enteral and parenteral nutrition used in this study contain amino acids, glucose, vitamins and minerals. Besides their function as precursors for protein synthesis, amino acids are able to replenish components of the tricarboxylic acid cycle which can increase ATP production in heart cells, with positive effects on cardiomyocytes metabolism [4]. Many of these amino acids are essential amino acids (histidine, isoleucine, leucine, lysine, methionine, phenylalanine, threonine, tryptophan, and valine) that cannot be synthesized by the human body and therefore need to be supplied by nutrition. The non-essential amino acids glutamate and aspartate are important compounds of nutrition since they are abundant intracellular free amino acids in the heart [24] which have been shown to be cardioprotective by enhancing ATP production [25]. Furthermore, in previous studies depleted levels of aspartate and glutamate in cardiomyocytes [26] and low plasma levels of arginine [27] have been found in patients with heart failure. Importantly, the semi-essential amino acid arginine is the precursor of NO, a dominant compound that influences blood flow and endothelial function, is involved in myocardial relaxation and distensibility, and might improve left ventricular function [7]. Furthermore, arginine supplementation might improve the arginine/ADMA ratio, an indictor of potential NO production.

The addition of glucose to (par)enteral nutrition can avoid conversion of the supplemented amino acids into glucose through gluconeogenesis, and can prevent protein catabolism [28]. Vitamins and minerals are essential ingredients of the nutrition because they prevent from micronutrient deficiency and they have antioxidant qualities [29].

## List of abbreviations used

ATP: adenosine triphosphate; NO: nitric oxide; NOS: nitric oxide synthase; ADMA: asymmetric dimethylarginine; CABG: coronary artery bypass grafting; FFM: fat free mass; AMC: Academic Medical Center; BIS: bio-impedance spectrometry; MPS: myocardial perfusion scintigraphy; ^18^F-FDG PET: ^18^F-fluorodeoxy-glucose positron emission tomography; SDMA: symmetric dimethylarginine; DDAH: dimethylarginine dimethylaminohydrolase; EuroSCORE: European System for Cardiac Operation Risk Evaluation score; ICU: intensive care unit; PBMC: peripheral blood mononuclear cells; SPECT: single-photon emission computed tomography; SSS: summed stress score; SRS: summed rest score; SDS: summed difference score; HPLC: high performance liquid chromatography; HE: hematoxylin and eosin; EVG: Elastica van Gieson; PAS/D: Periodic acid Schiff digested; MPO: myeloperoxidase; CML: carboxymethyl lysine; CK: creatine kinase.

## Competing interests

The authors declare that they have no competing interests.

## Authors' contributions

All authors: 1) have made substantial contribution to conception and design of the study; 2) have been involved in drafting the manuscript or revising it critically for important intellectual content; and 3) have given final approval of the version to be published.
